# Mood and Behavioral Changes in Adult Cerebral X-linked Adrenoleukodystrophy

**DOI:** 10.7759/cureus.117273

**Published:** 2026-09-30

**Authors:** Rohan Ram Setlur, Ranveer Singh, Manisha Jindal, Arpit Sharma

**Affiliations:** 1 Psychiatry, Armed Forces Medical College, Pune, IND; 2 Psychiatry, Command Hospital (Southern Command), Pune, IND

**Keywords:** adrenoleukodystrophy, mental disorders, mood disorders, secondary mood syndrome, white matter

## Abstract

A 33-year-old man with genetically confirmed X-linked adrenoleukodystrophy developed progressive irritability, emotional lability, anger outbursts, repetitive reassurance-seeking, and insomnia alongside worsening spastic paraparesis and urinary symptoms. Difficulty recalling familiar names and remembering and completing assigned tasks subsequently impaired occupational functioning. Collateral history established a departure from his calm and socially appropriate premorbid personality. Behavioral examination revealed overfamiliarity, disinhibited language, emotional lability, impaired judgment, and perseverative illness-related preoccupation. Cognitive examination demonstrated impaired attention, set-shifting, verbal fluency, inhibitory control, new learning, and recent recall, while neurological examination revealed pyramidal signs and a spastic gait. Brain magnetic resonance imaging showed symmetrical posterior cerebral white-matter hyperintensities, and genetic testing identified an ABCD1 c.323C>A variant. The temporal association with neurological progression, acquired behavioral change, cognitive impairment, and characteristic imaging abnormalities supported secondary mood syndrome with accompanying behavioral and cognitive manifestations rather than a primary depressive episode or adjustment disorder. Irritability, sleep, and anger outbursts improved during approximately one week of inpatient treatment with supportive intervention, low-dose quetiapine, and sodium valproate, although post-discharge follow-up was unavailable. This case emphasizes that new affective, behavioral, or cognitive symptoms in cerebral white-matter disease should prompt collateral history-taking, cognitive and neurological examination, and reassessment for disease progression.

## Introduction

X-linked adrenoleukodystrophy (X-ALD) is a peroxisomal disorder caused by pathogenic variants in ATP-binding cassette subfamily D member 1 (ABCD1), resulting in the accumulation of very-long-chain fatty acids and variable involvement of the cerebral white matter, spinal cord, peripheral nerves, adrenal cortex, and testes. Its clinical phenotype cannot be reliably predicted from genotype or plasma very-long-chain fatty-acid concentrations [1].

In adulthood, X-ALD commonly manifests as a slowly progressive myelopathy termed adrenomyeloneuropathy; cerebral demyelination may develop in approximately one-fifth of affected men with this phenotype [1,2]. Cognitive, behavioral, or affective changes may accompany or precede unmistakable neurological deterioration and can initially be attributed to a primary psychiatric disorder or an understandable psychological response to the diagnosis [3-6]. This creates a form of diagnostic overshadowing in which evidence of organic neuropsychiatric progression may be obscured by a seemingly plausible psychiatric explanation.

We describe an adult with genetically confirmed X-ALD whose evolving affective symptoms initially appeared consistent with psychological adjustment to a life-changing diagnosis. Collateral evidence of acquired behavioral change, disinhibition, and cognitive impairment, together with progressive pyramidal signs and characteristic cerebral white matter abnormalities, instead supported a secondary neuropsychiatric syndrome associated with cerebral disease.

## Case presentation

A 33-year-old male was diagnosed with X-linked adrenoleukodystrophy (X-ALD) after presenting with progressive tightening and weakness of both legs, urinary urgency with dribbling, recurrent headache, and erectile difficulty of four years’ duration. Genetic testing confirmed the diagnosis; next-generation sequencing detected an ABCD1 c.323C>A variant. He was referred for psychiatric evaluation seven months later, during hospitalization for scheduled follow-up, when ward staff had observed sudden crying and argumentative behavior. He had no previous psychiatric disorder, no relevant family history, and consumed alcohol occasionally.

He had functioned adequately at home and work before neurological onset. Following the diagnosis, his spouse noticed that he was unusually irritable, emotionally sensitive, and less predictable. By the next month, he had abrupt crying episodes disproportionate to the context and unexpected anger outbursts. Over the next two months, conversations repeatedly returned to prognosis, perceived unfairness of his illness, and possible transmission to his son, despite explanations and reassurance. He was specifically counseled that an affected father does not transmit an X-linked ABCD1 variant to his son. However, he remained preoccupied with the possibility and continued to request testing for reassurance. Initial insomnia developed as he spent prolonged periods thinking about the illness. These symptoms increased from a few episodes each week to almost daily approximately two months before the index admission. During the same period, lower-limb weakness, tripping, gait unsteadiness and urinary symptoms progressed.

By this point, colleagues noticed that he could not recall the names of familiar people and required supervision because he had difficulty remembering and completing assigned tasks. While awaiting his son’s genetic test result, he became increasingly argumentative and used overly familiar and occasionally abusive language that was alien to his premorbid behavior. Reassurance after the child’s normal result was short-lived. During hospitalization for scheduled review, he received a video depicting a head-injury survivor, which prompted repetitive concern that a minor head injury eight years earlier, rather than X-ALD, explained his symptoms. He repeatedly sought reassurance, cried frequently, expressed that life was not worth living, and behaved aggressively towards ward staff. Passive death wishes and behavioral disturbance prompted transfer to the psychiatry ward. There was no history of sustained elevated mood, panic episodes, intrusive thoughts, psychotic symptoms, or substance intoxication. His spouse and co-workers described his premorbid personality as cheerful, calm, patient, and sociable, with stable family and occupational relationships.

Neurological examination revealed intact cranial nerves. Both lower limbs had increased tone and Medical Research Council grade 4/5 power, brisk knee reflexes, ill-sustained ankle clonus, and extensor plantar responses. Gait was spastic with scissoring; sensory and cerebellar examinations were unremarkable.

Mental status examination revealed a kempt and cooperative patient who intermittently held the examiner’s hands, used overly familiar and obscene expressions, gestured excessively, and became tearful. Speech was spontaneous, verbose, coherent, and relevant. Affect was anxious, reactive, and labile. Thought content comprised illness-related preoccupation, helplessness, passive death wishes, and perseveration regarding the old head injury; delusions, obsessions, and perceptual abnormalities were absent. He was fully oriented with a clear sensorium.

Attention and concentration were impaired, with digit span forward/backward scores of 3/2 and one correct serial-seven subtraction. Immediate and recent memory and social and personal judgment were impaired, while remote memory, abstraction, and test judgment were preserved. Insight into psychiatric morbidity was absent. Biodrives were deranged in the form of reduced sleep with initial insomnia.

His 17-item Hamilton Depression Rating Scale score was 17 [7]. Bedside cognitive examination demonstrated impaired set-shifting, verbal fluency, and inhibitory control, along with impaired new learning and recent recall. This was a qualitative bedside assessment rather than a comprehensive neuropsychological evaluation.

Brain magnetic resonance imaging performed on a 1.5-T scanner showed bilaterally symmetrical, confluent T2-weighted/fluid-attenuated inversion recovery hyperintensities involving the periventricular, deep, and subcortical white matter of the parietal, occipital, and temporal lobes (Figures 1A, 1B). The posterior body and splenium of the corpus callosum and the corticospinal tracts extending to the pontomedullary junction were also involved. There was no evidence of cerebellar or spinal cord involvement, diffusion restriction, susceptibility-related signal changes, or post-contrast enhancement. Retrospective radiological scoring yielded a baseline Loes MRI severity score of 10/34 [8].

**Figure 1:**
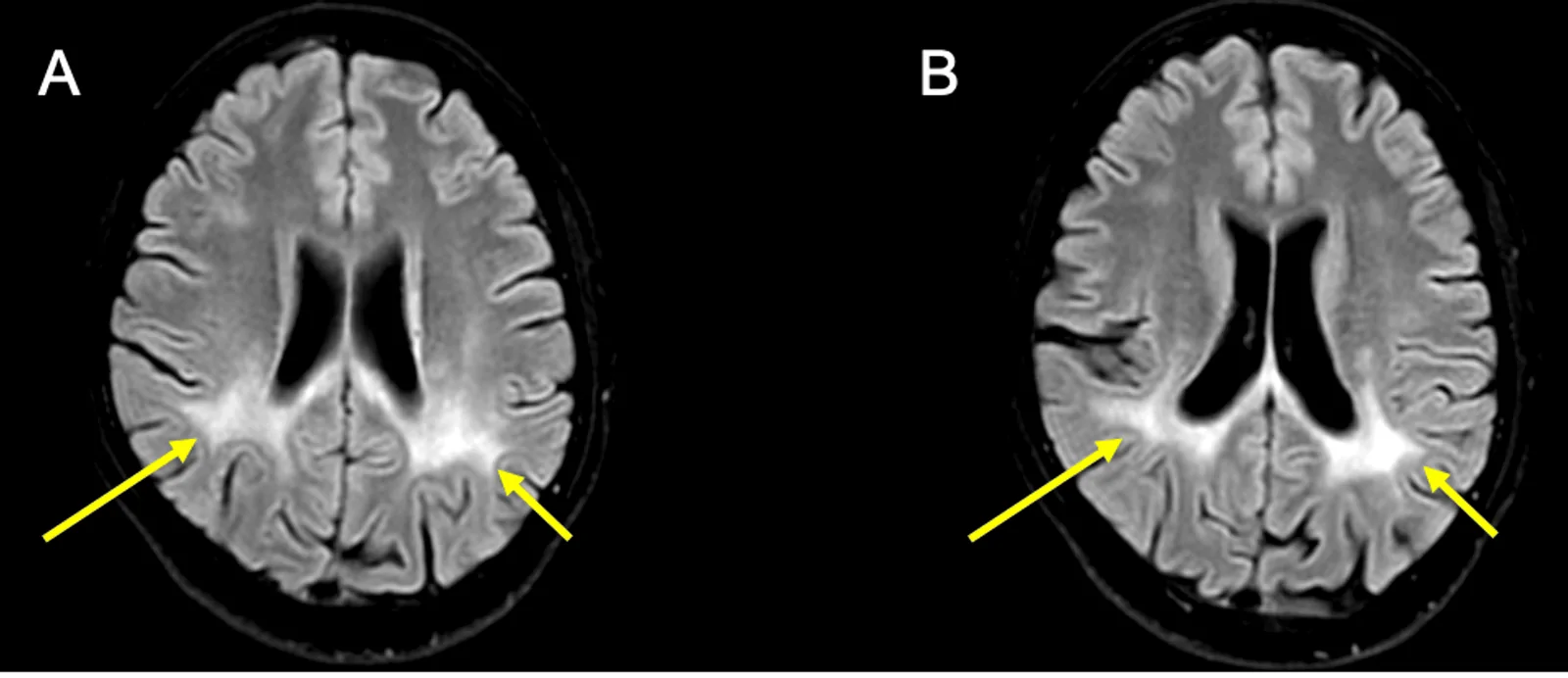
Axial fluid-attenuated inversion recovery magnetic resonance images demonstrating symmetrical posterior white-matter involvement. Axial fluid-attenuated inversion recovery images acquired on a 1.5-T scanner at two anatomical levels demonstrate symmetrical confluent hyperintensity in the bilateral parieto-occipital periventricular white matter (arrows) (A and B). Retrospective radiological assessment yielded a Loes MRI severity score of 10/34. No post-contrast enhancement was identified on the contrast-enhanced sequences (not shown).

Serum testosterone was low at 2.45 ng/mL, with raised luteinizing hormone at 12 mIU/mL, consistent with primary hypergonadotropic hypogonadism. Baseline morning cortisol and adrenocorticotropic hormone concentrations were not suggestive of adrenal insufficiency.

The concurrent neurological progression, acquired personality and behavioral change, disinhibition, cognitive dysfunction, pyramidal signs, imaging abnormalities, and confirmed ABCD1 variant supported secondary mood syndrome (International Classification of Diseases, 11th Revision; 6E62), rather than a moderate depressive episode or adjustment disorder [9].

Suicide risk was assessed, and he was observed serially. Psychoeducation and coping-focused supportive intervention were provided to the patient and his family. After a brief course of benzodiazepines, quetiapine 25 mg nightly and sodium valproate 250 mg nightly were started for behavioral dyscontrol, affective lability, and sleep disturbance. Baclofen and nutritional supplements prescribed by neurology and monthly testosterone prescribed by endocrinology were continued.

During approximately one week following initiation of medication, his irritability, sleep, and anger outbursts improved significantly. Excessive sedation and worsening of gait were not observed during this period. He was subsequently discharged home. Formal post-discharge follow-up was unavailable; therefore, the persistence of improvement and longer-term tolerability could not be assessed. The timeline of progression is depicted in Table 1.

**Table 1:** Clinical timeline showing neurological deterioration alongside emerging affective, behavioral, and cognitive symptoms, followed by psychiatric assessment, treatment, and short-term inpatient response.

Period	Key developments
7 months prior to admission	X-linked adrenoleukodystrophy was diagnosed following progressive spastic paraparesis, urinary symptoms, and erectile difficulty. Irritability and behavioral change began around the same time.
5 to 6 months prior to admission	Abrupt crying, anger outbursts, insomnia, illness-related preoccupation, and repetitive reassurance-seeking emerged.
2 months prior to admission	Symptoms became almost daily. Difficulty recalling familiar names and completing assigned tasks impaired occupational functioning and necessitated supervision.
Day 0	Disinhibition, aggression, and passive death wishes prompted psychiatric admission. Cognitive impairment and pyramidal signs were documented.
Inpatient days 1-7	Secondary mood syndrome with accompanying behavioral and cognitive manifestations was formulated. Supportive intervention, quetiapine, and sodium valproate were initiated. Irritability, sleep disturbance, and anger outbursts improved without observed excessive sedation or worsening gait. Follow-up data after discharge were unavailable.

## Discussion

The patient’s central diagnostic problem was attribution. Sadness, worry, and insomnia after a progressive genetic diagnosis can represent a psychologically mediated response; rating-scale evidence of depressive symptoms does not by itself establish a primary depressive disorder. Here, the more discriminating findings were a corroborated change from premorbid personality, overfamiliar and obscene language, emotional lability, perseveration, loss of occupational competence, executive and memory deficits, and bilateral pyramidal signs. Their evolution in parallel with neurological deterioration and parieto-occipital white-matter disease favored a direct neuropsychiatric consequence of X-ALD.

The diagnostic formulation nevertheless required differentiation among overlapping secondary syndromes. The disinhibition, altered interpersonal behavior, and impaired social judgment supported secondary personality change, while the executive and memory deficits and occupational decline supported a secondary neurocognitive syndrome. However, the emotional lability, recurrent crying, irritability, helplessness, insomnia, and passive death wishes were the symptoms that prompted psychiatric transfer and required immediate clinical intervention. The presentation was therefore formulated as secondary mood syndrome, with accompanying behavioral and cognitive manifestations, rather than as an isolated primary depressive episode or adjustment disorder [9].

Adult X-ALD may manifest as adrenomyeloneuropathy, cerebral demyelination, adrenal dysfunction, or overlapping phenotypes. Cerebral disease develops in approximately one-fifth of men with adrenomyeloneuropathy, although the proportion in whom psychiatric symptoms specifically precede neurological manifestations has not been reliably established [1,2]. Adult cerebral ALD can present with disinhibition, emotional lability, irritability, perseveration, psychosis, or cognitive decline [3-6,10]. Previous reports describe patients treated for chronic psychosis or mood disorder before spasticity, hyperreflexia, and white-matter abnormalities disclosed the underlying disease [3-5]. Shamim and Alleyne similarly described an adult whose psychiatric manifestations preceded the recognition of neurological disease by several years [11].

The index patient differed because X-ALD had already been diagnosed before psychiatric referral. The risk was therefore not failure to recognize the underlying neurological disorder, but diagnostic overshadowing in the opposite direction as follows: the affective and behavioral symptoms could be regarded solely as understandable psychological adjustment to a serious diagnosis while evidence of cerebral neuropsychiatric progression was overlooked. Psychological distress and organic progression were not mutually exclusive. Collateral history, evidence of acquired disinhibition, bedside cognitive examination, and the parallel progression of pyramidal signs helped distinguish the secondary neuropsychiatric syndrome from adjustment disorder.

The clinical picture integrated cerebral, myelopathic, and endocrine manifestations. Behavioral change, cognitive impairment, and the symmetrical posterior white-matter abnormalities supported cerebral involvement, whereas progressive spastic paraparesis, urinary symptoms, pyramidal signs, and a spastic gait supported myelopathic involvement. A normal spinal cord MRI does not exclude adrenomyeloneuropathy, which remains a clinical diagnosis [1]. The low testosterone with elevated luteinizing hormone was suggestive of primary hypergonadotropic hypogonadism. Baseline morning cortisol and adrenocorticotropic hormone were not suggestive of adrenal insufficiency; however, an adrenocorticotropic hormone stimulation test was not performed, and early adrenal dysfunction could therefore not be definitively excluded.

The symmetrical posterior cerebral white-matter pattern was compatible with cerebral ALD [1,8]. The Loes MRI severity score of 10/34 provided a standardized baseline measure of the anatomical burden of cerebral disease [8]. The absence of post-contrast enhancement indicated that no actively enhancing inflammatory margin was demonstrated at that examination, but it does not eliminate the need for continued surveillance. Adult men with X-ALD require longitudinal neurological assessment and annual brain MRI surveillance. Gadolinium is particularly indicated when a new or equivocal lesion is identified, or active cerebral disease is otherwise suspected [1,2]. New behavioral or cognitive symptoms should also prompt repeat neurological and neurocognitive assessment because cerebral disease may progress and eligibility for disease-modifying intervention is stage-dependent [1,2].

Evidence guiding psychotropic treatment in cerebral ALD is limited. In this case, low-dose quetiapine was selected to address sleep disturbance, affective lability, and behavioral dyscontrol while limiting extrapyramidal risk, and sodium valproate was selected for mood stabilization and impulsive aggression. An antidepressant was not initially selected because the presentation was not dominated by a sustained primary depressive syndrome. Psychotropics should be prescribed cautiously in cerebral white-matter disease because sedation, cognitive dulling, anticholinergic burden, postural instability, extrapyramidal effects, and metabolic adverse effects may compound gait impairment, fall risk, and neurological disability [2,3]. The observed improvement during one week of inpatient treatment was encouraging but cannot establish sustained efficacy.

Management should follow an integrated pathway combining neurology, adult psychiatry, endocrinology, and clinical genetics, with neuropsychological assessment where available and supportive care for the patient and family [1,2]. Psychiatric treatment may reduce distress and behavioral disturbance, but should not delay neurological surveillance, endocrine evaluation, genetic counseling, or consideration of disease-modifying treatment where appropriate.

This case is limited by bedside rather than comprehensive neuropsychological assessment and lack of formal post-discharge follow-up. Repeat symptom-rating scores were also unavailable; therefore, the short-term inpatient improvement is reported qualitatively and cannot establish treatment efficacy or longer-term tolerability. Nevertheless, the case illustrates a transferable diagnostic principle as follows: affective, behavioral, or cognitive change in white-matter disease warrants careful evaluation for neurological progression and secondary neuropsychiatric causes.

## Conclusions

Adult cerebral X-linked adrenoleukodystrophy may produce prominent affective, behavioral, and cognitive changes that resemble a primary psychiatric disorder or psychological adjustment to illness. In this patient, the parallel progression of mood and behavioral symptoms, cognitive impairment, spastic paraparesis, and characteristic cerebral white-matter abnormalities supported secondary mood syndrome with accompanying behavioral and cognitive manifestations. Assessment and management should integrate collateral history, neurological and cognitive examination, neuroimaging, endocrine evaluation, and genetic findings within coordinated neurological, psychiatric, endocrine, genetic, and family supportive care. New personality, behavioral, or cognitive change in a patient with cerebral white-matter disease should prompt reassessment for neurological progression before the presentation is attributed solely to a primary psychiatric disorder or psychological adjustment.

## References

[1] Engelen M, van Ballegoij WJ, Mallack EJ (2022). International recommendations for the diagnosis and management of patients with adrenoleukodystrophy: a consensus-based approach. Neurology.

[2] Kornbluh AB, Baldwin A, Fatemi A (2024). Practical approach to longitudinal neurologic care of adults with X-linked adrenoleukodystrophy and adrenomyeloneuropathy. Neurol Genet.

[3] Rosebush PI, Garside S, Levinson AJ, Mazurek MF (1999). The neuropsychiatry of adult-onset adrenoleukodystrophy. J Neuropsychiatry Clin Neurosci.

[4] Garside S, Rosebush PI, Levinson AJ, Mazurek MF (1999). Late-onset adrenoleukodystrophy associated with long-standing psychiatric symptoms. J Clin Psychiatry.

[5] Galvão AC, Machado-Porto GC, Porto FH, Lucato LT, Nitrini R (2012). Adult-onset adrenoleukodystrophy presenting as a psychiatric disorder: MRI findings. Dement Neuropsychol.

[6] Smith J, Williams MT, Misra VK (2018). Psychiatric disease in an adolescent as a harbinger of cerebral X-linked adrenoleukodystrophy. Pract Neurol.

[7] Hamilton M (1960). A rating scale for depression. J Neurol Neurosurg Psychiatry.

[8] Loes DJ, Hite S, Moser H (1994). Adrenoleukodystrophy: a scoring method for brain MR observations. AJNR Am J Neuroradiol.

[9] (2024). Clinical Descriptions and Diagnostic Requirements for ICD-11 Mental, Behavioural and Neurodevelopmental Disorders. https://iris.who.int/bitstream/handle/10665/375767/9789240077263-eng.pdf.

[10] Pierpont EI, Isaia AR, McCoy E, Brown SJ, Gupta AO, Eisengart JB (2023). Neurocognitive and mental health impact of adrenoleukodystrophy across the lifespan: insights for the era of newborn screening. J Inherit Metab Dis.

[11] Shamim D, Alleyne K (2017). X-linked adult-onset adrenoleukodystrophy: psychiatric and neurological manifestations. SAGE Open Med Case Rep.

